# Comorbidity in lung cancer patients and its association with hospital readmission and fatality in China

**DOI:** 10.1186/s12885-021-08272-y

**Published:** 2021-05-17

**Authors:** Dawei Zhu, Ruoxi Ding, Yong Ma, Zhishui Chen, Xuefeng Shi, Ping He

**Affiliations:** 1 China Center for Health Development Studies, Peking University, Beijing, 100191 China; 2 China Health Insurance Research Association, Beijing, 100013 China; 3 Department of Medical Insurance, Peking University Cancer Hospital and Institute, Key Laboratory of Carcinogenesis and Translational Research (Ministry of Education), Beijing, 100142 China; 4 School of Management, Beijing University of Chinese Medicine, Beijing, 100029 China

**Keywords:** Lung cancer, Comorbidity, China, Urban

## Abstract

**Background:**

Comorbidity has been established as one of the important predictors of poor prognosis in lung cancer. In this study, we analyzed the prevalence of main comorbidities and its association with hospital readmission and fatality for lung cancer patients in China.

**Methods:**

The analyses are based on China Urban Employees’ Basic Medical insurance (UEBMI) and Urban Residents’ Basic Medical Insurance (URBMI) claims database and Hospital Information System (HIS) Database in the Beijing University Cancer Hospital in 2013–2016. We use Elixhauser Comorbidity Index to identify main types of comorbidities.

**Results:**

Among 10,175 lung cancer patients, 32.2% had at least one comorbid condition, and the proportion of patients with one, two, and three or more comorbidities was 21.7, 8.3 and 2.2%, respectively. The most prevalent comorbidities identified were other malignancy (7.5%), hypertension (5.4%), pulmonary disease (3.7%), diabetes mellitus (2.5%), cardiovascular disease (2.4%) and liver disease (2.3%). The predicted probability of having comorbidity and the predicted number of comorbidities was higher for middle elderly age groups, and then decreased among patients older than 85 years. Comorbidity was positively associated with increased risk of 31-days readmission and in-hospital death.

**Conclusion:**

Our study is the first to provide an overview of comorbidity among lung cancer patients in China, underlines the necessity of incorporating comorbidity in the design of screening, treatment and management of lung cancer patients in China.

## Background

As one of the most commonly diagnosed malignancy and the greatest cause of cancer-related death worldwide, lung cancer has imposed a considerable challenge to global public health [1]. It is estimated that approximately 0.8 million new cases occurred in 2018, accounting for 18.1% of newly diagnosed cancer [2], and the five-year survival rate was only 17.8% [3], much lower than that of other leading cancers like colorectal cancer or stomach cancer [2]. The situation is even worse in China, which contains 19% of the world population but with more than one-third of newly diagnosed lung cancer patients and 37.6% of lung cancer deaths worldwide [4]. Lung cancer has been the leading cause of cancer mortality in China for decades, accounting for almost 20% of total deaths [5]. In 2018, the estimated age-standardized mortality rate for lung cancer in China was 30.9 per 100,000 persons [2, 6], greatly higher than the world average (18.6 per 100,000), [2, 7]. According to the Global Burden of Disease study [8], the disease burden of lung cancer continues to be substantial throughout the world within the foreseeable future. In China, the incidence and mortality of lung cancer was predicted to climb at a more rapid rate than that in western countries in the next decade [6].

The diagnosis of lung cancer is associated with advanced age and smoking, both of which are related to the occurrence of concomitant chronic diseases [9, 10]. Comorbidity has been established as one of the key predictors of poor prognosis in different cancers [11]. It potentially affects lung cancer survival by influencing the choice of antineoplastic treatment [12] and by camouflaging the cancer symptoms and causing diagnosis delay [13]. Comorbidity may also deteriorate patients’ performance status often more than the tumor development [14]. Therefore, epidemiologic studies on comorbidity of lung cancer is very important for its assessment and management, health service resources, as well as the interpretation of cancer statistics [15]. However, despite the increasing recognition of the impact of comorbidity on lung cancer treatment and prognosis from western studies, little research has investigated comorbidities in lung cancer patients in China, leaving the prevalence and main types, as well as the impact of concomitant conditions unknown, not to mention the urgent need in such a country with rapid population aging, air pollution and a large smoking population [5].

Using health insurance claims data to obtain medical information in patients has been demonstrated to be an efficient and valid method for the identification of comorbidities in cancer populations [16, 17]. Comparing with the inaccuracy and limitation of self-reported information [18], the comorbid conditions in such administrative databases were generally recorded by professional diagnosis, offering an alternative to improve quality of related epidemiological research without requiring additional prospective registration efforts [16].

Using claims data from China Urban Employee’s Basic Medical Insurance (UEBMI) and China Urban Residents’ Basic Medical Insurance (URBMI), this study aims to analyze the prevalence of main comorbidities and its association with hospital readmission and fatality for lung cancer patients in urban population, which accounted for more than 55% of new cases and lung cancer deaths in China [5]. It will provide a starting point for future studies on the impact of comorbidity on lung cancer treatment and prognosis in China.

## Methods

### Data source

The first part analyses were based on the claims data of random sample of UEBMI and URBMI held by the China Health Insurance Research Association (CHIRA), and it is corresponding to about 5% of the total urban beneficiary. The sample was drawn using systematic random sampling strategy with a random start. In brief, every Kth record from a population of size N was selected, with the first sample record picked from a random number table. In such a way, a sample size of n was obtained, where N/n > =K. In urban China, UEBMI and URBMI are two major health insurance programs covered more than 93% of the residents in the urban area [19]. The database includes 65 cities in 2013 to 2016, and contains all the records of urban beneficiaries’ demographic information and primary diagnoses of hospital admissions and outpatient visits. Since the claims data were collected for accounting purpose, there are only 23 cities reported the second and other diagnosis. We excluded 10 cities, in which the sample proportion less than 1% and 5 cities with the number of lung cancer patients less than 500. Of the 8 remaining cities, 7 of them were province capitals. To maintain sample consistency, we selected seven provincial capitals (Beijing, Hangzhou, Fuzhou, Wuhan, Changsha, Chengdu, and Kunming).

In the second part analyses, we collected additional data form Hospital Information System (HIS) Database in the Beijing University Cancer Hospital from January 2016 to March 2018. Beijing University Cancer Hospital was established in 1976, and it has been one of the top cancer hospitals in China. It has 1040 health professionals and 790 hospital beds, with 450 thousand outpatient visits and 40 thousand inpatients admissions in 2013. The HIS database contains diagnostic and basic socio-demographic information.

### Measurements

In both insurance claims data and hospital information system data, we used the ICD-10 (the 10th revision of the International Statistical Classification of Diseases) to identify lung cancer patients with principal diagnosis codes C34. Diagnosis of lung cancer was made by qualified clinical practitioners according to Chinese Guidelines on the Diagnosis and Treatment of Primary Lung Cancer (2011 version to 2016 version). Strictly and comprehensive general clinical examination as well as endoscopy and pathological test were the cardinal diagnosis techniques.

We obtained information on comorbid conditions using secondary and other diagnosis. Diagnosis of secondary and other disease was also made by qualified special physicians. There are many scales to measure comorbidity, such as Charlson Comorbidity Index, Adult Comorbidity Evaluation, Elixhauser Comorbidity Index, etc. [20, 21]. According to the comorbidity character of our sample, we use Elixhauser Comorbidity Index (contains 31 types of comorbidities) to assess comorbidity, which has been demonstrated to be a more comprehensive and better predictive tool for outcomes of people with cancer [22], and for large Chinese electronic medical record databases [23].

Comorbidity was defined as the presence of one or more of 31 comorbidities in Elixhauser index excepting lung cancer. And according to the number of comorbidity, it was further categorized into four categories: 0, 1, 2 and ≥ 3. In addition, we classified main comorbidities into six groups: other malignancy (lymphoma, metastatic cancer, solid tumor without metastasis), hypertension (uncomplicated and complicated), pulmonary disease (pulmonary circulation disorders, chronic pulmonary disease), diabetes mellitus (uncomplicated and complicated), cardiovascular disease (congestive heart failure, cardiac arrhythmias, vascular disease) and liver disease.

Hospital readmission is defined as patient’s admission to a hospital within 31 days after being discharged from an earlier hospital stay. Death of an admitted patient which occurs in hospital was treated as in-hospital death. Demographic characteristics included age groups (younger than 45 years, 45–49 years, 50–54 years, 55–59 years, 60–64 years, 65–69 years, 70–74 years, 75–79 years, 80–84 years, and 85 years or older) and sex.

### Ethical approval

Since the claims data we used was an anonymized database and no influence on patient care, the Institutional Review Board of Peking University Health Science Centre deemed this study as exempt from ethical approval.

### Patient and Public Involvement

In the first part analysis, we extracted a 5% random sampling from claims data of China Urban Employees’ Basic Medical Insurance (UEBMI) and Urban Residents’ Basic Medical Insurance (URBMI) from 2013 to 2016, which covered more than 93% of residents in urban China. The data, which was collected by China Health Insurance Research Association (CHIRA), included 65 cities and contains beneficiaries’ demographic information, medical diagnoses and expenditures of outpatient and inpatients services. 10,175 anonymized patients with lung cancer were identified in the dataset between 2013 and 2016.

In the second part analyses, we collected additional data form Hospital Information System (HIS) Database in the Beijing University Cancer Hospital from January 2016 to March 2018. 20,902 anonymized patients with lung cancer were identified in this dataset.

### Statistical analysis

Descriptive analysis was used to analyze the sample characteristics. We conducted frequency analysis to examine comorbidity distribution in the sample. Logistic and Poisson regression were used to investigate the association between age groups and the presence of comorbidities and the number of comorbidities, respectively, and predicted comorbidity probabilities and the number of comorbidities were used to illustrate these associations. Logistic regression was used to evaluate the contribution of the comorbidities to predicting 31-day readmission and in-hospital death.

Control variables included in the models were sex, insurance type, city, and year. We performed logistic regression to investigate the relationship between age groups and 6 selected six conditions (listed above). In addition, we tested whether these relationships differ by sex using the interaction between age groups and sex.

A *p*-value of less than 0.05 was considered statistically significant. The software Stata version 15 for Windows (Stata Corp, College Station, TX, USA) was used for the statistical analysis.

## Results

### Sample characteristics

10,175 patients with lung cancer were identified in the claims data between 2013 and 2016. The median age was 65 years, 60.4% of patients was male, and 77.6% of the sample was covered by UEMBI in the claims data between 2013 and 2016 (Table 1). The sample characteristics of HIS data was shown in Table 4 in Appendix.

**Table 1:** Sample characteristics (Claims data)

	2013 (*n* = 3271)	2014 (*n* = 3529)	2015 (*n* = 1524)	2016 (*n* = 1851)	Total (*n* = 10,175)
Age					
0–44, n(%)	231 (7.1)	177 (5.0)	79 (5.2)	85 (4.6)	572 (5.6)
45–49, n(%)	182 (5.6)	172 (4.9)	51 (3.3)	77 (4.2)	482 (4.7)
50–54, n(%)	251 (7.7)	318 (9.0)	148 (9.7)	159 (8.6)	876 (8.6)
55–59, n(%)	482 (14.7)	496 (14.1)	178 (11.7)	170 (9.2)	1326 (13.0)
60–64, n(%)	478 (14.6)	603 (17.1)	234 (15.4)	293 (15.8)	1608 (15.8)
65–69, n(%)	425 (13.0)	555 (15.7)	235 (15.4)	224 (12.1)	1439 (14.1)
70–74, n(%)	396 (12.1)	410 (11.6)	217 (14.2)	216 (11.7)	1239 (12.2)
75–79, n(%)	376 (11.5)	412 (11.7)	191 (12.5)	191 (10.3)	1170 (11.5)
80–84, n(%)	181 (5.5)	266 (7.5)	109 (7.2)	132 (7.1)	688 (6.8)
85+, n(%)	269 (8.2)	120 (3.4)	82 (5.4)	304 (16.4)	775 (7.6)
Sex					
Female, n(%)	1296 (39.6)	1438 (40.7)	573 (37.6)	687 (37.1)	3994 (39.3)
Male, n(%)	1975 (60.4)	2091 (59.3)	951 (62.4)	1164 (62.9)	6181 (60.7)
Health insurance					
URBMI, n(%)	734 (22.4)	412 (11.7)	250 (16.4)	338 (18.3)	1734 (17.0)
UEBMI, n(%)	2537 (77.6)	3117 (88.3)	1274 (83.6)	1513 (81.7)	8441 (83.0)

#### Comorbid conditions among lung cancer patients in urban China

Table 2 presents the prevalence of the comorbid conditions by sex among lung cancer patients from claims data. 32.2% of the sample had at least one comorbid condition, and the proportion of patients with one, two, and three or more comorbidities was 21.7, 8.3 and 2.2%, respectively. The most prevalent comorbidities were other malignancy, hypertension, pulmonary disease, diabetes mellitus, liver disease and cardiovascular disease, with prevalence of 7.5, 5.4, 3.7, 2.5, 2.4 and 2.3%, respectively. Sex-specific results were also provided in Table 2.

**Table 2:** Prevalence of comorbid conditions by sex among lung cancer patients in urban China

	Female (*n* = 3994)	Male (*n* = 6181)	Total (n = 10,175)
Numbers of comorbidity			
0, n(%)	2746 (68.8)	4154 (67.2)	6900 (67.8)
1, n(%)	869 (21.8)	1336 (21.6)	2205 (21.7)
2, n(%)	309 (7.7)	539 (8.7)	848 (8.3)
3+, n(%)	70 (1.8)	152 (2.5)	222 (2.2)
Comorbidity			
Other malignancy, n(%)	318 (8.0)	448 (7.2)	766 (7.5)
Hypertension, n(%)	238 (6.0)	313 (5.1)	551 (5.4)
Pulmonary disease, n(%)	71 (1.8)	309 (5.0)	380 (3.7)
Diabetes mellitus, n(%)	97 (2.4)	154 (2.5)	251 (2.5)
Liver disease, n(%)	124 (3.1)	118 (1.9)	242 (2.4)
Cardiovascular disease, n(%)	87 (2.2)	150 (2.4)	237 (2.3)

**Fig. 1:**
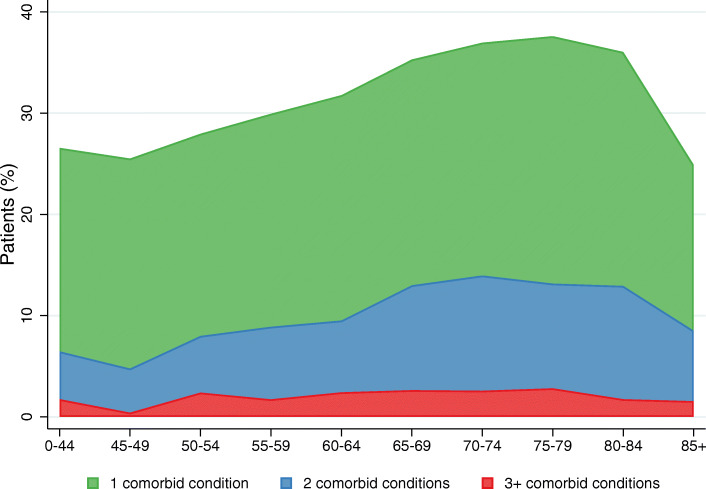
Proportion of having different numbers of comorbid conditions among lung cancer patients in China. Shows the proportion of having one, two and three or more comorbid conditions among lung cancer patients in urban China. (Green color: 1 comorbid condition; blue color: 2 comorbid conditions; red color: 3+ comorbid conditions)

Figure 1 displays the prevalence of having one, two and three or more comorbidities among different age groups of lung cancer patients. The prevalence of having one or more, two or more, three or more comorbid diseases peaked at the age groups of 75 to 79 years, 70 to 74 years, 75 to 79 years, with prevalence of 37.6, 14.0 and 2.8%, respectively.

#### Association between age and comorbidities among Lung cancer patients in urban China

**Fig. 2:**
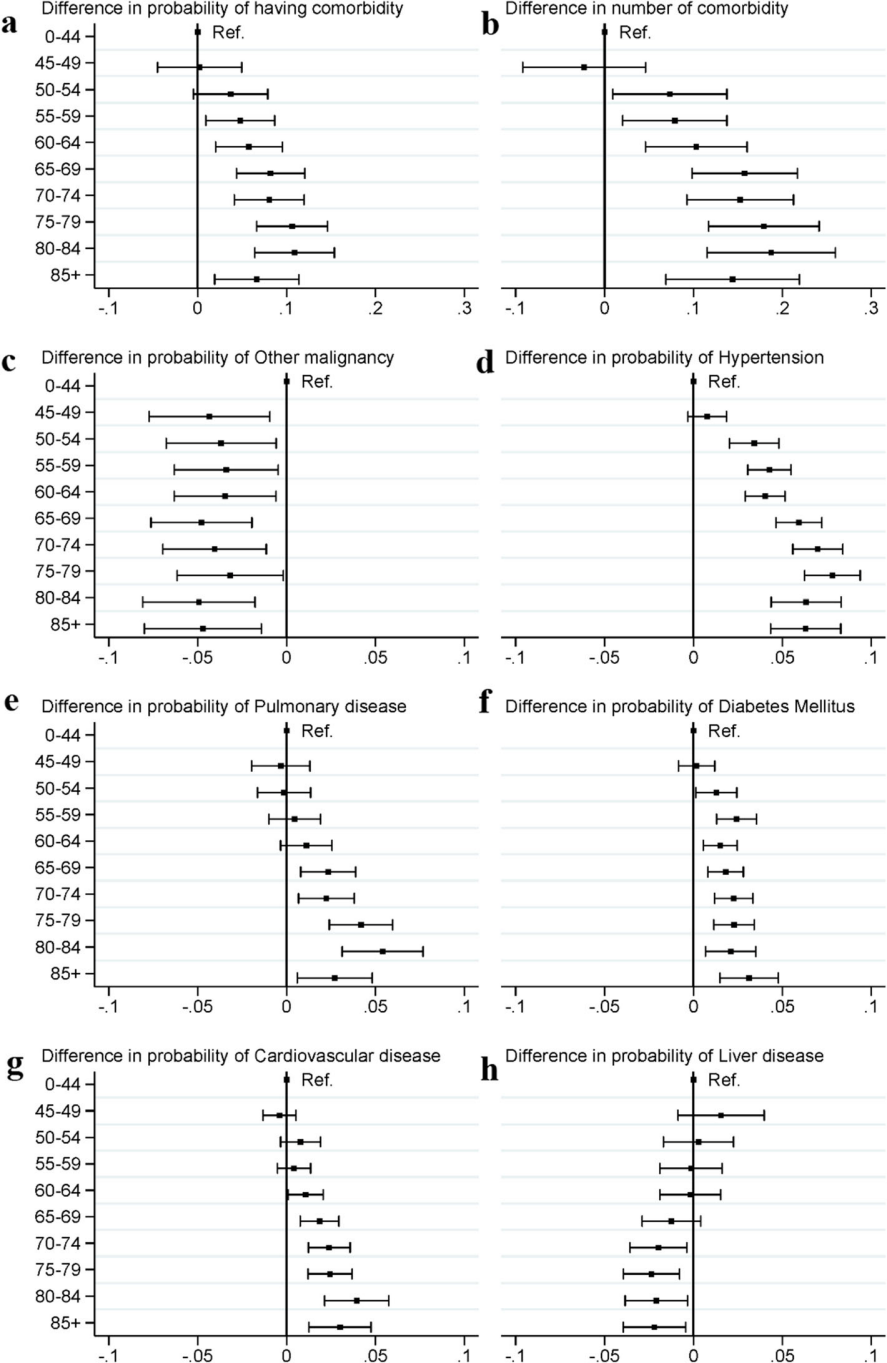
Difference and 95%CI in probability of comorbid conditions between age groups (reference group: 0–44 years) among lung cancer patients in urban China. **a** shows the difference in predicted probability (95% CI) of comorbidities between age groups (reference group: 0–44 years) among lung cancer patients in urban China. **b** shows the difference in predicted number (95% CI) of comorbidities between age groups (reference group: 0–44 years) among lung cancer patients in urban China. **c** shows the difference in predicted probability (95% CI) of having other malignancy between age groups (reference group: 0–44 years) among lung cancer patients in urban China. **d** shows the difference in predicted probability (95% CI) of having hypertension between age groups (reference group: 0–44 years) among lung cancer patients in urban China. **e** shows the difference in predicted probability (95% CI) of having pulmonary disease between age groups (reference group: 0–44 years) among lung cancer patients in urban China. **f** shows the difference in predicted probability (95% CI) of having diabetes mellitus between age groups (reference group: 0–44 years) among lung cancer patients in urban China. **g** shows the difference in predicted probability (95% CI) of having cardiovascular disease between age groups (reference group: 0–44 years) among lung cancer patients in urban China. **h** shows the difference in predicted probability (95% CI) of having liver disease between age groups (reference group: 0–44 years) among lung cancer patients in urban China

Fig. 2a shows that after adjusting for sex, health insurance type, city and year, the difference in the predicted probability of having comorbidity between different age groups.

The predicted probability of having comorbidity increased with age among lung cancer patients, with 10.9% (95%CI, 6.4, 15.4%) higher of probability in the group of 80–84 years compared to the group of 0–44 years. Similar pattern was also observed in the predicted number of comorbidities (Fig. 2b), with relatively higher predicted number in patients aged 65 to 84 years, and a decline in 85+ years age groups.

Figure 2 c-h presents the association between age groups and six major type of comorbidities among lung cancer patients. For hypertension (75–79 years: 7.8, 95%CI: 6.3, 9.4%), pulmonary disease (80–84 years: 5.4, 95%CI: 3.1, 7.7%), diabetes mellitus (85+ years: 3.1, 95% CI: 1.5, 4.8%) and cardiovascular disease (80–84 years, 3.9, 95%CI:2.1, 5.8%), the predicted probability was higher among patients in older age groups compared to the group of 0–44 years. And the predicted probability of hypertension, pulmonary disease and cardiovascular disease started to decline from the age groups of 80 to 84 years, 85+ years and 85+ years, respectively. But the result of other malignancy and liver disease showed reversed patterns, with lower predicted probability in older age groups

### Sex-difference of comorbidity in different age groups of lung cancer patients in urban China

**Fig. 3:**
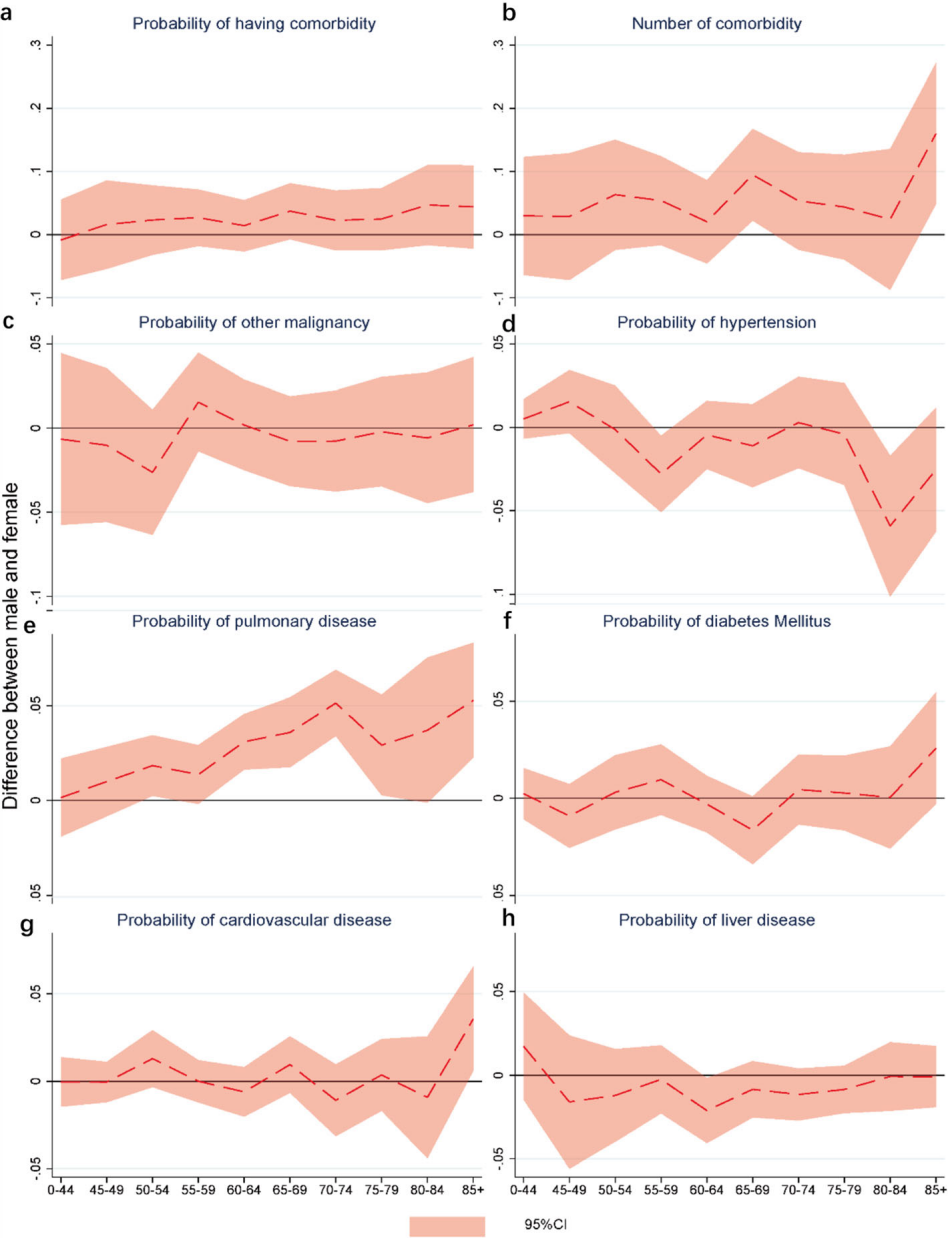
Sex difference in the probability of comorbid conditions by age groups among lung cancer patients in urban China. **a** shows the sex difference of the predicted probability (95% CI) of comorbidities by age groups among lung cancer patients in urban China. **b** shows the sex difference in predicted number (95% CI) of comorbidities by age groups among lung cancer patients in urban China. **c** shows the sex difference of the predicted probability (95% CI) of having other malignancy by age groups among lung cancer patients in urban China. **d** shows the sex difference of the predicted probability (95% CI) of having hypertension by age groups among lung cancer patients in urban China. **e** shows the sex difference of the predicted probability (95% CI) of having pulmonary disease by age groups among lung cancer patients in urban China. **f** shows the sex difference of the predicted probability (95% CI) of having diabetes mellitus by age groups among lung cancer patients in urban China. **f** shows the sex difference of the predicted probability (95% CI) of having cardiovascular disease by age groups among lung cancer patients in urban China. **h** shows the sex difference of the predicted probability (95% CI) of having liver disease by age groups among lung cancer patients in urban China

As Fig. 3a displayed, there was no significant sex-difference in the probability of having comorbidity conditions in all age groups. However, for the age groups of 65–69 years and 85+ years, the respective predicted number of comorbidities (Fig. 3b) was 0.09 (95% CI: 0.02, 0.17) and 0.16 (95%CI: 0.05, 0.27) higher among male patients comparing with those among females.

The sex difference in the risk of having six main types of comorbidity in different age groups was provided in Fig. 3c-h. Male patients aged 60–79 years and 85+ years had significantly higher probability of comorbid pulmonary disease comparing with female patients in the same age groups. And for patients aged 85+ years, the probability of having concurrent cardiovascular disease was 3.6% (95%CI: 0.1, 6.6%), which was higher for males than that for females. In contrast, the risk of having hypertension was found to be higher among female patients aged 55–59 years and 80–84 years, as well as the risk of liver disease in the age group of 60–64 years. No significant difference of predicted probability in other type of comorbid conditions was found between male and female samples.

### Association between comorbidity and readmission and in-hospital death among lung cancer patients in urban China

The 31-day readmission rate for claims data and HIS data were 67.6 and 38.9%, respectively and the in-hospital death for HIS data was 0.2%. Table 3 displays the result of logistic regression analysis on the association between comorbidity and readmission and in-hospital death among lung cancer patients. Both the results of analysis on claims data (OR: 1.75, 95%CI: 1.68, 1.83) and HIS data (OR: 2.31, 95%CI: 2.13, 2.51) showed that the risk of 31-days readmission was significantly higher among patients having comorbidity compared to that among their counterparts without any comorbid conditions. And increased number of comorbidities was also associated with increased risk of 31-days readmission among lung cancer patients. In the association between comorbidity and in-hospital death, a significantly higher risk of in-hospital death (OR: 8.77, 95%CI: 1.10, 70.18) was only observed among lung cancer patients with three or more comorbidities in the result of analysis on HIS data.

**Table 3:** Association between comorbidity and in-hospital death and readmission (OR, 95%CI)

	Claim data^a^ (n = 10,175)	Hospital Information System^b^ (n = 20,902)	
	31-day readmission	31-day readmission	In-hospital death
Having comorbility	1.751 (1.676,1.830)	2.309 (2.129,2.505)	2.936 (0.382,22.549)
Numbers of comorbidity (Ref. = 0)			
1	1.429 (1.353,1.510)	2.064 (1.893,2.250)	0.658 (0.065,6.665)
2	2.062 (1.905,2.231)	2.578 (2.346,2.834)	1.934 (0.222,16.880)
3+	3.901 (3.411,4.463)	2.706 (2.438,3.004)	8.770 (1.096,70.177)

Note: ^a^, claim data of 5% random sample of China Urban Employee’s Basic Medical Insurance and China Urban Residents’ Basic Medical Insurance; ^b^, hospital information system database in the Beijing University Cancer Hospital

## Discussion

This study, using claim of urban health insurance, is the first to provide the most recent situation on comorbidity in lung cancer patients in urban China. Our study identified the main comorbidities and estimated the age- and sex-specific prevalence of comorbidity, as well as its association with hospital readmission and fatality in Chinese urban lung cancer patients. The comparison of comorbidity patterns with existing literature from developed countries were illustrated below.

The prevalence of comorbidities for lung cancer patients in urban China was lower than that in developed countries. Studies found that 43.3% of patients with lung cancer had at least one comorbidities in Sweden [24] and 87.3% in Scotland [25]. Nevertheless, different prevalence of comorbidity across studies may be caused by the heterogeneity of data source, including the difference in selection, detection and classification [26]. One study in lung cancer patients indicated the significant difference of comorbidity prevalence between self-reported and register-based data [27]. In addition, medical information system in China has yet to be improved [28], and thus the inaccuracy and incompleteness of patients’ medical information may also result in low comorbidity prevalence. Meanwhile, we found that the median age of Chinese lung cancer patients (65 years old) was younger than that of the counterparts (> = 69) in developed nations [18, 29]. This may further explain the relatively low prevalence of comorbidity since advanced age was considered as a key predictor of developing concomitant chronic diseases [9]. Nevertheless, investigation based on improved claims data set is guaranteed in the future to provide more comprehensive and accurate estimation on comorbidity among lung cancer patients in China.

In our study, comorbidities most commonly identified were other malignancy, hypertension, pulmonary disease, diabetes mellitus, cardiovascular diseases and liver disease, which were generally consistent with both international studies [14, 16] and localized research [30, 31] in China. However, we found that the most prevalent comorbidity was other malignancy in Chinese lung cancer patients, instead of pulmonary diseases in most of other literatures, which may suggest the delayed diagnosis due to the relatively inadequate prevention and screening services in China. Approximately 75% of lung cancer patients was in advanced stage at the time of diagnosis [32] in China, significantly higher than that in developed countries, ranging from 47.9% in Sweden to 64.9% in Australia [33].

Similar to the patterns from previous western studies in both lung cancer and other cancer patients [26, 34, 35], the overall and most types of comorbidity among Chinese lung cancer patients increased in prevalence and numbers across the early elderly age spectrum, plateaued during middle elderly age (70–84), and began to decline with advanced age (85+). Higher prevalence and severity of comorbidity among older groups was relatively self-evident since aging is a process that accompanied with ever-increasing susceptibility to disease and death. And the decline in the advanced age group may be explained by the natural attrition of those lung cancer patients with comorbidity before reaching 85+ years [35]. Nevertheless, the age pattern of certain type of comorbidity, such as other malignancy and liver disease, showed reversed trends. Given a limited theoretical explanation for this issue, further research is required to explore the in-depth causation. These patterns have implications for the design of screening questionnaires and functional assessments, as well as the development of treatment and research protocol to address the prevalent comorbidity for lung cancer patients from different age groups [35].

Our study also found the difference in both the prevalence and predicted probability of pulmonary diseases and cardiovascular diseases between male and female samples. This could be explained by the fact that cigarette smoking is by far the most significant risk factors for lung cancer [26]. It is estimated that there are 350 million smokers in China, and they consumed approximately 30% of world tobacco every year [36]. Most of the Chinese smokers were men. The smoking rate (60%) was much higher in males than that in females in China(< 5%) [37]. A body of literature [38–40] demonstrated that chronic respiratory diseases were more commonly occurred among men and patients with squamous-cell carcinoma, both of which contains highest proportions of smokers. And other health risk behaviors like high-glucose-high-fat diet and alcohol drinking, was found to be more prevalent in smokers. This may also contribute to the higher prevalence of cardiovascular diseases among male lung cancer patients [41].

The significant association between comorbidity and 31-days readmission and in-hospital death among lung cancer patients in China was in line with previous research. Studies from United States [42] and Ireland [43] suggested that both having comorbidity and the number of comorbidities were positively associated with re-hospitalization rate among lung cancer patients. And several research [12, 14, 25] from western countries also demonstrated comorbidity as a highly significant predictor of reduced survival. Generally, cancer patients with comorbidities always suffer from more severe health conditions. Besides the independent prognostic effect of comorbidity, during the past decade, increased treatment options for lung cancer has made marked improvement in patients’ survival [44], but for those with comorbidities, their potential and performance towards aggressive antineoplastic treatment or therapeutic clinical trials may be detrimentally affected, and further resulted in relatively poor prognosis [25].

Our study has several limitations. First, we used cross-sectional data, which means that the development of new comorbid ailments after the time of diagnosis cannot be considered. However, the impact was relatively minor considering the relatively short survival time of lung cancer patients in China. Secondly, we are unable to investigate the prevalence of comorbidity according to histological type and stage of lung cancer, as well as the cause of in-hospital death due to a lack of related information in the data source. Thirdly, extrapolation from our finding to the comorbid situation of lung cancer patients in China should be cautious because the claims database we used was restricted to urban population of seven cities. However, currently, these cities were the few sites that reported relatively complete information about comorbidity among lung cancer patients in China. Fourthly, our result is subject to potential bias since dementia is not included in the Elixhauser Comorbidity Index, and we also cannot estimate the nutritional status (obesity or weightless) of lung cancer patients, which were identified as comorbidities in Elixhauser Index, since related information was not available in the dataset. Last but not the least, lung cancer was considered as the index disease in collecting information on comorbidity, so that the prevalence and number of comorbidity may be underestimated due to the exclusion of the cases that consider lung cancer as the comorbidity and other diseases as index disease. And as we mentioned before, the incompleteness of patients’ medical records may also further lead to the underestimation of the prevalence. The main strength of our study is using medical insurance claims data, which was universally covered urban residents in China [19], to identify patients with comorbidities. The medical insurance record is generally regarded as relatively complete and valid source of information on the patient’s health status and thus provide an opportunity for us to investigate the situation of comorbidity among lung cancer patients in urban China.

## Conclusion

In conclusion, our study first provides an overview of comorbidity among lung cancer patients in urban China, contributes to the world literature in developing nations of a non-Western context. Our result suggests that for future research and related policy formulation, comorbidity situation should be incorporated with screening promotion, treatment choice, quality of life, duration of survival and research on clinical and psychosocial interventions for Chinese lung cancer patients.

## Data Availability

The data were provided by China Health Insurance Research Association. These are third party data. Authors in this study have the right to use this dataset, but not the right to share and distribute. A de-identified minimal dataset of the quantitative data is available upon request to researchers who meet the criteria for confidential information, by sending a request to phe@pku.edu.cn.

## Appendix

**Table 4:** Sample characteristics of Hospital Information System (HIS) Data (*n* = 20,902)

	No. of patients, n (%)
Age	
0–44	1271 (6.08)
45–49	1609 (7.70)
50–54	3220 (15.41)
55–59	3664 (17.53)
60–64	5117 (24.48)
65–69	3270 (15.64)
70–74	1796 (8.59)
75–79	766 (3.66)
80–84	160 (0.77)
85+	29 (0.14)
Sex	
Female	6762 (32.35)
Male	14,140 (67.65)
Health insurance	
UEBMI	14,022 (67.08)
Others	6880 (32.92)

*UEBMI* Urban Employees’ Basic Medical insurance

## Abbreviations

UEBMI: Urban Employees’ Basic Medical insurance;
URBMI: Urban Residents’ Basic Medical Insurance;
HIS: Hospital Information System;
CHIRA: China Health Insurance Research Association;
ICD-10: the 10th revision of the International Statistical Classification of Diseases;
CI: Confidence Interval;
OR: Odds Ratio
